# 

**Published:** 1891-12-19

**Authors:** 


PRICE ONE PENNY.

[No.] 273, Vol. XI.—December 19th, 1891.

[Registered at the General Post Office as a Newspaper for transmission in the United Kingdom and Abroad.]

WITH EXTRA NURSING SUPPLEMENT.
Per Annum, 6s. 6d., post free.
Monthly Parts, 6d.; post free, 8d.
Ditto per Annum, 8s., post free.

### THE HOSPITAL

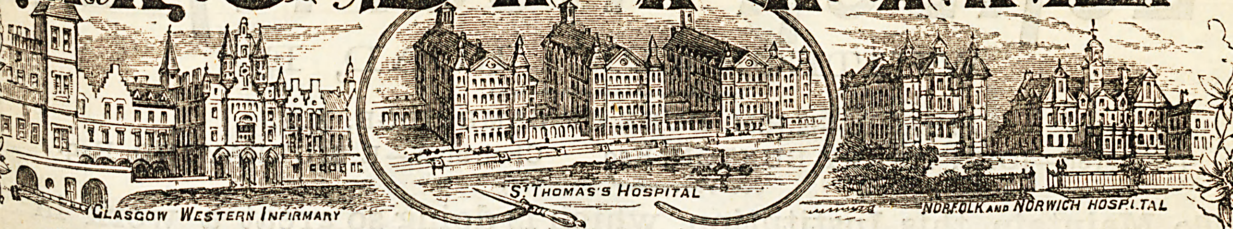

A WEEKLY JOURNAL OF

### Science, Medicine, Nursing, and Philanthropy.

### CHRISTMAS APPEAL NUMBER.

SATURDAY, DECEMBER 19th, 1891.

London Without Hospitals . . . . . 133

The Princess of Wales and her Son . . . . . 140

Everybody's Page . . . . . 140

Christmas at the Hospitals . . . . . 141

Notes and News . . . . . 142

Passing Topics.—Proportionate Giving . . . . . 142

Sweet Charity's Guide for Christmas Givers.—Being the Christmas Number of The Hospital—General Hospitals, 143; Special Hospitals, 145; Nursing Institutions—A Few Other Charities . . . . . 147

New Drugs, Appliances, and Things Medical. — An excellent Bronchitis Kettle—Salvine Dentifrice . . . 148

EXTRA SUPPLEMENT.—THE NURSING MIRROR.

En Passant.—A Cure for Hysteria—Guild of St. Veronica—A New Home at Glasgow—Tenby Tittle-Tattle—Short Items—Glasgow Sick Poor Nursing Association . . . . xlvii

Lectures on Surgical Ward Work and Nursing. By Alex. Miles, M.D. Edin., F.R.C.S.E. Lecture XXXIX. —Needles and Trephining Instruments . . . . lxviii

For Reading to the Sick—The Fading Year . . . lxix

Everybody's Opinion — Notice of Appointment — Sick Nurses—St. Mary's Cottage Hospital—A Pupil from Cyprus—"Moral Bacilli" . . . . lxxi

Our Indian Letter . . . . . lxx

Appointments . . . . . lxxi

Amusements and Relaxation . . . . . lxxi

Off Duty.—The Lay of a Combatant—The Lay of a Non-Combatant . . . . . lxxii

Christmas Competitions . . . . . lxxii

Presentation . . . . . lxxii

Death in our Ranks . . . . . lxxii

Wants and Workers . . . . . lxxii

Notes and Queries . . . . . lxxii

